# Protective Effects of Intralipid and Caffeic Acid Phenethyl Ester on Nephrotoxicity Caused by Dichlorvos in Rats

**DOI:** 10.1155/2015/491406

**Published:** 2015-10-04

**Authors:** Muhammet Murat Celik, Ayse Alp, Recep Dokuyucu, Ebru Zemheri, Seyma Ozkanli, Filiz Ertekin, Mehmet Yaldiz, Abdurrahman Akdag, Ozlem Ipci, Serhat Toprak

**Affiliations:** ^1^Department of Internal Medicine, Medical Faculty, Mustafa Kemal University, 31000 Hatay, Turkey; ^2^Department of Biochemistry, The Government Hospital of Obstetrics and Gynecology, 31000 Hatay, Turkey; ^3^Department of Medical Physiology, Medical Faculty, Mustafa Kemal University, 31000 Hatay, Turkey; ^4^Department of Pathology, Medeniyet University Goztepe Training and Research Hospital, 81054 Istanbul, Turkey; ^5^Department of Internal Medicine, Ministry of Health Batman Regional Government Hospital, 72000 Batman, Turkey; ^6^Department of Medical Pathology, Medical Faculty, Mustafa Kemal University, 31000 Hatay, Turkey; ^7^Department of Chemistry, Science and Arts Faculty, Mustafa Kemal University, 31000 Hatay, Turkey

## Abstract

The protective effects of Caffeic Acid Phenethyl Ester (CAPE) and intralipid (IL) on nephrotoxicity caused by acute Dichlorvos (D) toxicity were investigated in this study. Forty-eight Wistar Albino rats were divided into 7 groups as follows: Control, D, CAPE, intralipid, D + CAPE, D + IL, and D + CAPE + IL. When compared to D group, the oxidative stress index (OSI) values were significantly lower in Control, CAPE, and D + IL + CAPE groups. When compared to D + IL + CAPE group, the TOS and OSI values were significantly higher in D group (*P* < 0.05). When mitotic cell counts were assessed in the renal tissues, it was found that mitotic cell count was significantly higher in the D group while it was lower in the D + CAPE, D + IL, and D + IL + CAPE groups when compared to the control group (*P* < 0.05). Also, immune reactivity showed increased apoptosis in D group and low profile of apoptosis in the D + CAPE group when compared to the Control group. The apoptosis level was significantly lower in D + IL + CAPE compared to D group (*P* < 0.05) in the kidneys. As a result, we concluded that Dichlorvos can be used either alone or in combination with CAPE and IL as supportive therapy or as facilitator for the therapeutic effect of the routine treatment in the patients presenting with pesticide poisoning.

## 1. Introduction

Organophosphorus pesticides (OPs) have been widely and effectively used for applications in agricultural settings, public health, commerce, and individual households worldwide in order to increase efficiency of agricultural production and maintain hygienic conditions [1, 2]. Dichlorvos (2, 2-dichlorovinyl phosphate) (D) is an OP that is widely used worldwide. Since its commercial introduction in 1961, D has been increasingly used in many countries and produced important benefits by controlling internal and external parasites in livestock and domestic animals as well as insects in houses and fields [3]. However, the extensive applications of D inevitably cause environmental, soil, and crop pollution. Consequently, human exposure to low levels of D became chronic via contaminated food and water. Recently, the effects of D on human health have raised increasing attention in community [4]. The clinical signs and symptoms associated with acute D poisoning are generally attributable to acetylcholine (ACh) accumulation following the inhibition of acetylcholinesterase (AChE). Overstimulation of the ACh causes the clinical signs and symptoms including muscarinic, nicotinic, and central nervous system toxic effects [5]. In addition, acute cholinergic effects may cause irreversible and progressive neurological deficits in both humans and animals [6].

Several antidotes have been evaluated for the routine treatment of OP poisoning and the currently recommended drugs are atropine and pralidoxime chloride [7]. Atropine has been used as antidote against OPs over past decades, as it effectively antagonizes the muscarinic receptors, but not nicotinic receptor, against toxic effects of Ach [5]. Some studies have demonstrated that D has toxic effects such as hepatotoxicity, renal toxicity, and neurotoxicity. However, new methods and drug investigations are needed for support or protective clinical treatment against nephrotoxicity caused by OP toxication.

Recently, oral IL emulsion was introduced as a novel method in the treatment of intoxication from several lipophilic agents. Since it was shown to be effective in bupivacaine toxicity, IL may be a promising approach for other lipophilic drug intoxications, including herbicides and pesticides. Moreover, it has been suggested that IL binds lipophilic agents and confines liposoluble toxic elements. It is also reported that it is administered by a bolus dose of 1.5 mL/kg and an infusion dose of 0.25 mL/kg/min in liposoluble drugs (clomipramine, propranolol, bupropion, haloperidol, and organophosphates). However, IL is not currently used in the treatment protocols because of insufficient evidence in intoxications [8].

Caffeic acid phenethyl ester (CAPE) is a compound that is structurally similar to the flavonoid found in bee propolis. It is an active component of propolis extract, which is one of the reactive oxygen species (ROS) that occurs as a result of oxidative stress in toxic failure and ischemia-reperfusion injury and has tissue protective effect [9]. CAPE reaches to required blood concentration when administered intraperitoneally [10] and CAPE, at 10 mmol/kg concentration, inhibits the xanthine oxidase system and the formation of ROS [9, 11].

This study concluded that CAPE and IL are capable to exert protective effects against nephrotoxicity caused by acute D toxicity in rats. Therefore, this study shed light on the literature in terms of the prophylactic use of these two agents in the disorders induced by oxidative damage and distant organs damage.

## 2. Materials and Methods

### 2.1. Animals, Care, and Nutrition

The study was approved (ethic number: 2012-065) by Necmettin Erbakan University, Experimental Medical Research Center's Experimental Animals Ethics Committee, Konya, Turkey. The forty-eight mature Wistar Albino rats weighing 200–250 g were randomly divided into seven groups. The animals were kept under laboratory conditions of 12-hour light-dark cycle at a room temperature (21°C ± 2°C).

### 2.2. Animals and Treatment

The forty-eight rats were randomly divided into seven groups (*n* = 6) as Control (C), Dichlorvos (*n* = 7), intralipid (*n* = 7), CAPE (*n* = 7), Dichlorvos + intralipid (*n* = 7), Dichlorvos + CAPE (*n* = 7), and Dichlorvos + intralipid + CAPE (*n* = 7) groups. Doses and durations of medication were adjusted according to the literature so the rats were given Dichlorvos (4 mg/kg/day via oral route) [3, 12], intralipid (18.6 mL/kg via oral route) [8, 13], and CAPE (10 *μ*mol/kg via intraperitoneal route) [10, 11]. IL and CAPE were administered immediately after D administration as single dose. Rats were sacrificed under ketamine/xylazine (90/10 mg/kg) anesthesia. Kidney tissues of the rats that were removed were stored at −70°C for biochemical analysis whereas a part of kidney specimen was kept in formaldehyde solution for histologic examination.

### 2.3. Biochemical Analysis

The TAS of supernatant fractions was evaluated by using a novel automated and colorimetric measurement method developed by Erel. Hydroxyl radicals, the most potent biological radicals, are produced in this method. In the assay, the ferrous ion solution present in reagent 1 is mixed with hydrogen peroxide, which is present in reagent 2. The radicals produced subsequently, such as brown-colored dianisidinyl radical cations produced by the hydroxyl radicals, are also potent radicals. Using this method, the antioxidative effect of the sample is measured against the potent-free radical reactions initiated by the produced hydroxyl radicals. The assay has excellent precision values (lower than 3%). The TAS results are expressed as nmol Trolox equivalent/mg protein. The TOS of supernatant fractions was also evaluated by using a novel automated and colorimetric measurement method developed by Erel. Oxidants present in the sample oxidize the ferrous ion-o-dianisidine complex to ferric ion. The oxidation reaction is amplified by glycerol molecules, which are abundantly present in the reaction medium. The ferric ion produces a colored complex with xylenol orange in an acidic medium. The color intensity, which can be measured spectrophotometrically, is related to the total amount of oxidant molecules present in the sample. The assay is calibrated with hydrogen peroxide, and the results are expressed in terms of nanomoles of H_2_O_2_ equivalent/milligram of protein. The units of TOS and TAS were micromoles of H_2_O_2_ equivalent/gram protein and millimole of H_2_O_2_ equivalents/gram protein, respectively. The Oxidative stress index (OSI) value was calculated as follows: OSI = TOS/TAS [14, 15]. Ellagic acid attenuates oxidative stress on brain and sciatic nerve and improves histopathology of brain in streptozotocin-induced diabetic rats [16].

### 2.4. Histopathologic Analysis

#### 2.4.1. Hematoxylin and Eosin Method

For histopathologic examinations, routine histologic paraffin block preparation method was used after fixation of kidney samples that are kept in 10% formalin. 5 *μ*m thick sections were obtained from paraffin blocks by using a microtome (Leica Rotary; Leica Microsystems GmbH, Wetzlar, Germany). Collected sections were stained with hematoxylin-eosin (H&E) and examined under light microscope (×100) and photos were captured. Histopathology of the tissue samples was rated semiquantitatively according to normal tissue composition. In the pathological examinations of the sample parameters of brush border loss, extravasation, tubular cast structures, nucleus loss in the tubule epithelial cells, tubular dilatation and interstitial accumulation of lymphocytes, and tubular necrosis were rated from 0 to 5 points; thus, normal kidneys and other applied kidneys were compared. This scoring (0: normal tissue, 1: blown tubular epithelium cell areas, vacuolar degradation, and necrosis, less than 25% of cases, 2: 25–50% of similar cases, 3: 50–75% of similar cases, 4: more than 75% of similar cases, 5: complete cortical necrosis) was carried out according to similar scoring of the other studies in kidney [17].

#### 2.4.2. Immunohistochemistry Method

Immunohistochemical examination was performed on a Leica Bond-Max automated IHC/ISH platform (Leica Microsystems Inc., Buffalo Grove, Illinois). Four-micrometer paraffin sections were dewaxed in a Bond Dewax solution and rehydrated in alcohol and Bond Wash solution (Leica Microsystems). Antigen retrieval was performed using a high pH (ER2) retrieval solution for 15 minutes followed by endogenous peroxidase blocking for 5 minutes on the machine. Anti-mouse monoclonal antibody Bcl-2 (C-2: sc-7382, Santa Cruz Biotechnology, Inc., in dilution 1 : 200), anti-mouse monoclonal antibody Bax (B-9: sc-7480, Santa Cruz Biotechnology, Inc., in dilution 1 : 100), and anti-mouse caspase-3 (CPP32) monoclonal antibody (clone JHM62, Leica Biosystems Ltd., Newcastle) were applied at 1 : 50 dilution for 60 minutes at room temperature. Detection was performed using the Bond Polymer Refine Red Detection system (Leica Microsystems) with a 15-minute postprimary step followed by 25-minute incubation with alkaline phosphatase-linked polymers. Sections were then counterstained with hematoxylin on the machine, dehydrated in alcohols, and mounted with mounting medium (Sakura Finetek USA, Inc., Torrance, California). Prepared tissues were observed by histopathologists blinded to the experimental study groups. The numbers of apoptotic cells were counted in ten randomly selected microscope fields under a ×400 magnification in a blind fashion. The average number of stained neurons for each set of ten fields was calculated and expressed as the number of the positive cells/high-power field.

### 2.5. Statistical Evaluation

SPSS 11.5 software was used for statistical analysis. Data were expressed as mean ± SD. Kruskal-Wallis test was applied to determine the abnormal distribution followed by post hoc Tukey's and Mann-Whitney *U* test. *P* < 0.05 was considered to be statistically significant.

## 3. Results

### 3.1. Biochemical Analysis

#### 3.1.1. TAS Levels

The TAS level was significantly lower in the D group than those in the other groups (*P* < 0.05). the TAS values were significantly higher in Control, CAPE, and D + IL  +  CAPE groups when compared to the D group (*P* < 0.05). Also the TAS values were significantly higher in the CAPE and IL groups when compared to the D + IL + CAPE group (*P* < 0.05) (Table 1).

#### 3.1.2. TOS Levels

The TOS values were significantly lower in the Control, IL, CAPE, D + CAPE, and D + IL + CAPE groups when compared to D group (*P* < 0.05), while no significant difference was observed among other groups (*P* > 0.05) (Table 1). Also, the TOS values were significantly higher in the D group compared to the D + IL + CAPE group (*P* < 0.05) (Table 1).

#### 3.1.3. OSI Levels

The OSI values were significantly lower in the Control, CAPE, and D + IL + CAPE group when compared to the D group (*P* < 0.05) while no significant difference was observed among other groups (Table 1). Also, the OSI values were significantly higher in the D group when compared to the D + IL + CAPE group (*P* < 0.05) (Table 1).

### 3.2. Histopathologic Results

**Figure 1:**
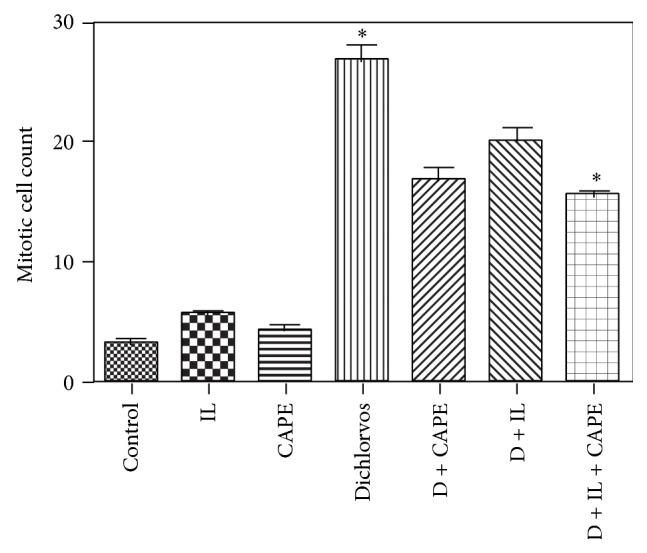
Histopathologic results for kidney tissues. Mitotic cell count. ANOVA test (intralipid (IL), Dichlorvos (D)).

**Figure 2:**
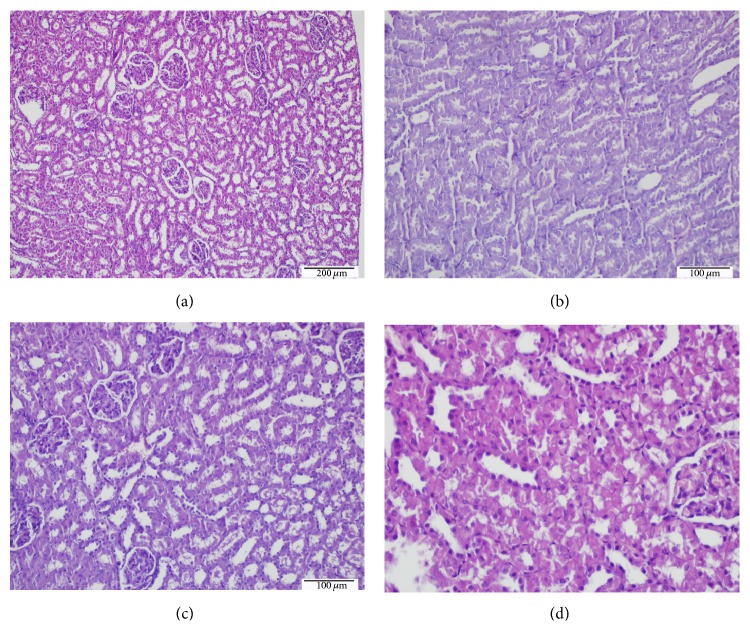
Evaluation of mitosis (hematoxylin and eosin method). (a) Control rats with normal renal histology (H&E, ×100). (b) Common mitotic increase in the rats with Dichlorvos (H&E, ×100) (D). (c) Decreasing mitotic density and regenerative changes in the rats given CAPE following Dichlorvos (H&E, ×200) (D + CAPE). (d) Relative decrease in the mitotic density and regenerative changes in the rats given CAPE and IL following Dichlorvos (H&E, ×200) (D + CAPE + IL).

When the mitotic counts were assessed in the renal tissues, it was found that mitotic count was higher in the D group (Figures 1 and 2(b)) and significantly lower in the D + CAPE (Figure 2(c)), D + IL, and D + IL + CAPE (Figure 2(d)) groups compared to the Control group (Figure 2(a)) (Figure 2, H&E) (Table 2).

**Figure 3:**
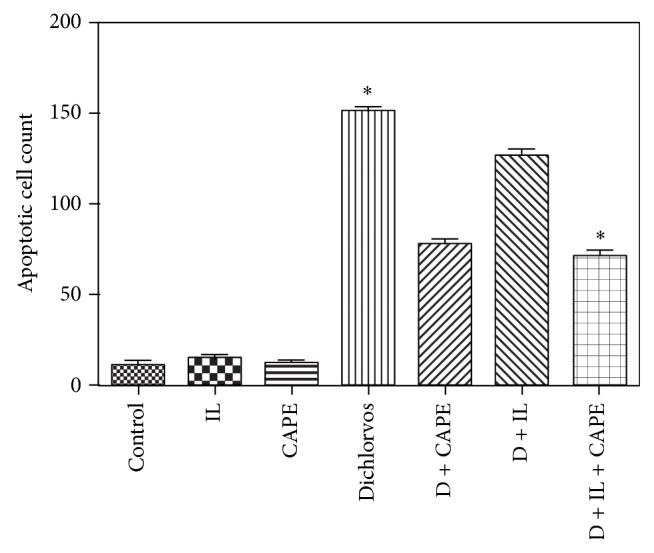
Histopathologic results for kidney tissues. Apoptotic cell count. ANOVA test (intralipid (IL), Dichlorvos (D)).

**Figure 4:**
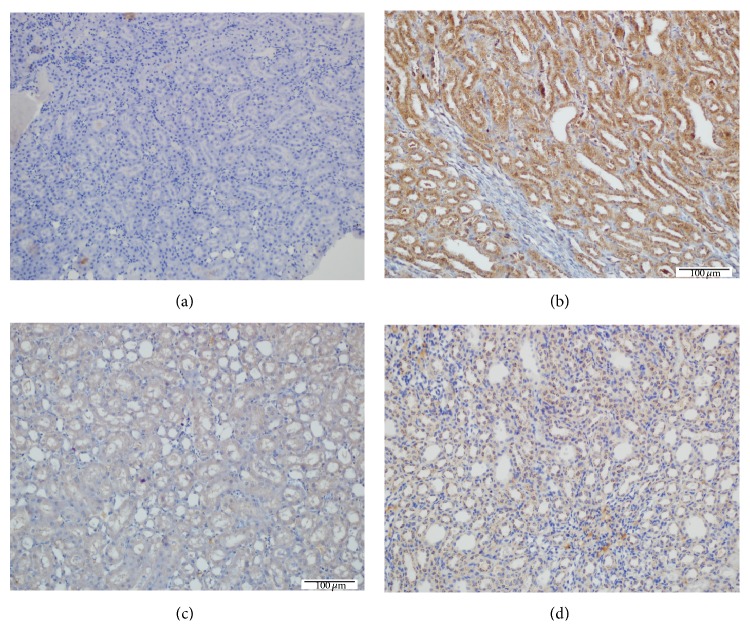
Evaluation of apoptosis by Bcl-2, Bax, and caspase-3 (immunohistochemical method). (a) Unusual development of immune reactivity in Control rats (IHC, ×200). (b) Immune reactivity shows increased apoptosis in the kidneys given Dichlorvos (IHK, ×200). (c) Low profile of apoptosis in the D + CAPE group (IHC, ×200). (d) The apoptosis level in the D + IL group is only lower than in the D group (IHC, ×400).

Using caspase-3, Bcl-2, and Bax, immune reactivity showed increased apoptosis in the kidneys from the D group (Figures 3 and 4(b)) and low profile of apoptosis in the D + CAPE group (Figure 4(c)). The apoptosis level was significantly lower in the D + IL + CAPE group (Figure 4(d)) than the D group (Figure 4). The apoptosis was evaluated in the renal cells and it was found that there were 10.2 ± 1.49 apoptotic cells in the Control group, 11.7 ± 1.49 in CAPE, 14.8 ± 2.54 in IL, 151.3 ± 6.49 in D, 78.8 ± 3.67 in D + CAPE, 127.4 ± 4.89 in D + IL, and 72.1 ± 3.02 in D + CAPE + IL. This indicated that the apoptosis rate was lower in the D + CAPE and D + CAPE  +  IL groups compared to the D group (Table 3, Figure 3).

## 4. Discussion

When organophosphate is absorbed through the skin or by means of digestion, mucosal membranes, conjunctiva, or respiration, organophosphate (OP) intoxication leads to quite a serious clinical picture, even leading to sudden onset of respiratory failure which requires admission to intensive care unit [18, 19]. OP has several toxic effects on other systems such as neurotoxicity, myocardial toxicity, embryotoxicity, hepatotoxicity, immunotoxicity, genetic toxicity, and nephrotoxicity [20, 21].

The oral acute LD50 dose of D, which is an OP compound, is 80 mg/kg in rats [3]. Previous studies showed that the application of D with a dose of 7.2 mg/kg leads to pathologic and biochemical changes in the renal cells [22]. Also, some other studies showed that an oral dose of 4 mg/kg in rats induces a decrease in sperm motility [3] and endometrial injury [12]. Therefore, the rats were administered with D (4 mg/kg/day via oral route) in the present study. Ben Amara et al. reported that OPs lead to kidney injury and increased malondialdehyde (MDA) levels, cystatin C levels, and plasma creatinine and uric acid levels and that these injuries can be ameliorated via vitamin E and selenium [23]. On the other hand, Silfeler et al. revealed that paraquat intoxication leads to pancreatic injury and that CAPE (10 *μ*mol/kg via intraperitoneal route) is effective in reversing this injury [11]. Therefore, the rats were given CAPE (10 *μ*mol/kg via intraperitoneal route) in the present study.

Similarly, Tuzcu et al. claim that IL diminishes the pancreatic injury caused by malathion, an OP, and that IL exerts these effects through decreasing malathion absorption by acting as a chelate with malathion in the stomach [8]. Similar to the above studies, in the D group of the present study, histopathologic analysis revealed significant nucleus loss in the tubule epithelial cells, tubular dilatation and interstitial accumulation of lymphocytes, and tubular necrosis. Quasi-normal renal tissue with blown tubular epithelium cell areas, vacuolar degradation, and necrosis less than 25% was observed in the D + CAPE and IL groups.

Toxic cell death is known to appear by only two main mechanisms: necrosis and apoptosis, which can be divided by their characteristic morphological and biochemical situations. These aspects of apoptosis are observed in various tissues from animal models, and while there is widespread necrosis occurring within the area of injury, apoptosis also plays an important role in toxic cell death in various tissues [24].

Hou et al. have demonstrated that rat exposure to D caused renal injury, including renal tubular, glomerular filtration, and oxidative stress. Also, these toxic effects were also regulated by high-dose quercetin. In that study, histopathological examination revealed that D induced extensive cell vacuolar denaturation; however, milder histopathological alterations were observed in the kidney tissues of rats by combined D + quercetin (50 mg/kg bw) [22]. Fiore et al. showed that D-induced apoptosis is mediated by pesticide's capacity to induce monopolar spindle-associated mitotic cell arrest, which, in turn, promotes apoptosis directly from mitosis [25]. In a study conducted by Alp et al., CAPE was demonstrated to be an effective agent in protection against injuries in liver, lungs, and kidney caused by diazinon which is an OP [26].

The results of above referred studies were in agreement with our results. In present study, the D group revealed cytoplasmic hypereosinophilic changes, loss of intracellular boundaries, nuclear pyknosis, diffusely increased mitotic activity in histopathologic examinations, and diffusely increased apoptosis in immunohistochemical analysis. However, in the rats given D + CAPE, D + IL, and D  +  IL  +  CAPE the mitotic density was decreased and regenerative changes were observed. Similarly, in the immunohistochemical analysis, apoptosis was increased in the D group while it was lower in D + CAPE, D + IL, and D  +  IL  +  CAPE groups compared to the D group. These results show that CAPE and IL may exclusively ameliorate the renal injury caused by D in the present study.

When biochemical results were evaluated in some studies, it was reported that OPs cause increase of oxidative stress, ROS, and depletion of antioxidant enzymes [27]. Celik and Suzek showed that the sublethal concentrations of D have toxic effects on MDA content and antioxidant defense system such as reduced glutathione (GSH), glutathione reductase (GR), superoxide dismutase (SOD), and glutathione-S-transferase (GST) in various tissues of rats exposed to 0.0225- and 0.0450-millimole (mmol) D in drinking water [28]. Also, Cao et al. showed that the activities of GST, SOD, and catalase (CAT) were decreased dramatically in the D-treated group when compared with the flavonoid extracts group and the D  +  flavonoid extracts group [29].

Our results were in agreement with abovementioned studies. The present study showed that the administration of D causes increase in the TOS levels and decrease in the TAS levels compared to the Control, IL, CAPE, D + CAPE, and D  +  IL  +  CAPE groups. Also, the TAS levels in D + CAPE, D + IL, and D + IL + CAPE groups were found higher than in the D group. In the present study, histopathological results were consistent with biochemical results.

Based on our results, it can be concluded that CAPE and IL are similarly capable of preventing the renal injuries caused by D by means of their antioxidant effects. Therefore, we suggest that it can be used either alone or in combination with CAPE and IL or it can be used as supportive therapy or as facilitator for the therapeutic effect of the routine treatment in the patients presenting with pesticide poisoning. However, further studies are needed about protective effects of CAPE and IL on OP intoxications.

## Figures and Tables

**Table 1:** Comparison of postmedication total antioxidant status (TAS), total oxidant status (TOS), and oxidative stress index (OSI) levels in renal tissues.

	TAS (mmol Trolox Eq./g protein)	TOS (mmol H_2_O_2 _Equiv./g protein)	OSI (H_2_O_2_/Trolox)
Control	1.50 ± 0.16	132.9 ± 16.71	88.35 ± 7.79
CAPE	1.49 ± 0.20	120.1 ± 30.36	93.2 ± 13.62
IL	1.30 ± 0.30	133.8 ± 9.55	107.5 ± 28.36
Dichlorvos	1.17 ± 0.08^a^	169.6 ± 8.61^b^	126.9 ± 13.24^c^
Dichlorvos + CAPE	1.31 ± 0.06	137.1 ± 24.87	104.6 ± 21.08
Dichlorvos + IL	1.33 ± 0.07	141.7 ± 25.41	106.3 ± 18.42
Dichlorvos + IL + CAPE	1.44 ± 0.12^d^	135.3 ± 21.19^e^	94.55 ± 18.41^f^

Data are presented as mean ± SD. Kruskal-Wallis and post hoc Tukey's and Mann-Whitney *U* tests were used. The mean difference is significant at the level of 0.05. (*P* < 0.05).

^a^Compared with the Dichlorvos group, the TAS values in the Control, CAPE, and D + IL + CAPE groups were significantly higher (*P* = 0.001).

^b^Compared with the Dichlorvos group, the TOS values in Control, IL, CAPE, D + CAPE, and D + IL + CAPE groups were significantly lower (*P* = 0.01).

^c^Compared with the Dichlorvos group, the OSI values in Control, CAPE, and D + IL + CAPE groups were significantly lower (*P* = 0.001).

^d^Compared with the Dichlorvos + IL + CAPE group, the TAS values in the Dichlorvos group were significantly lower (*P* = 0.001).

^e,f^Compared with the Dichlorvos + IL + CAPE group, the TOS and OSI values in the Dichlorvos group were significantly higher (*P* = 0.01).

D (Dichlorvos), IL (intralipid), and CAPE (caffeic acid phenethyl ester).

**Table 2:** Histopathologic results for kidney tissues, mitotic cell count.

	Mean ± SD	Min–Max
Control	3.1 ± 1.34	1–5
CAPE	4.4 ± 0.97	3–6
IL	5.5 ± 0.97	4–7
D	26.5 ± 3.99	20–32
D + CAPE	16.86 ± 2.41	13–20
D + IL	20.14 ± 2.61	16–24
D + IL + CAPE	15.43 ± 1.27	14–17

Control versus D, D + CAPE, D + IL, and D + IL + CAPE	*P* < 0.0001	
Dichlorvos versus D + CAPE, D + IL, and D + IL + CAPE	*P* < 0.0001	
CAPE versus D, D + CAPE, D + IL, and D + IL + CAPE	*P* < 0.0001	
IL versus D, D + CAPE, D + IL, and D + IL + CAPE	*P* < 0.0001	
D + IL versus D + IL + CAPE	*P* < 0.001	

Kruskal-Wallis and post hoc Tukey's and Mann-Whitney *U* tests were used.

**Table 3:** Histopathologic results for kidney tissues, apoptotic cell count.

	Mean ± SD	Min–Max
Control	10.2 ± 1.49	8–12
CAPE	11.7 ± 1.49	10–14
Intralipid (IL)	14.8 ± 2.54	11–18
Dichlorvos (D)	151.3 ± 6.49	140–160
D + CAPE	78.8 ± 3.67	75–85
D + IL	127.4 ± 4.89	119–134
D + IL + CAPE	72.1 ± 3.02	68–76

Control versus D, D + CAPE, D + IL, and D + IL + CAPE	*P* < 0.0001	
Dichlorvos versus D + CAPE, D + IL, and D + IL + CAPE	*P* < 0.0001	
CAPE versus D, D + CAPE, D + IL, and D + IL + CAPE	*P* < 0.0001	
IL versus D, D + CAPE, D + IL, and D + IL + CAPE	*P* < 0.0001	
D + IL versus D + IL + CAPE	*P* < 0.0001	
D + CAPE versus D + IL + CAPE	*P* < 0.01	

Kruskal-Wallis and post hoc Tukey's and Mann-Whitney *U* tests were used.
